# Heart failure events in randomized controlled trials for adults receiving maintenance dialysis: a meta-epidemiologic study

**DOI:** 10.1093/ndt/gfae156

**Published:** 2024-07-10

**Authors:** David Collister, Lonnie Pyne, Arrti A Bhasin, Brendan Smyth, William Herrington, Meg Jardine, Patrick B Mark, Sunil Badve, Patrick Rossignol, Laura M Dember, Christoph Wanner, Justin Ezekowitz, P J Devereaux, Patrick Parfrey, Ron Gansevoort, Michael Walsh

**Affiliations:** Division of Nephrology, Department of Medicine, University of Alberta, Edmonton, Canada; Population Health Research Institute, Hamilton, Canada; Canadian Vigour Center, Edmonton, Canada; Population Health Research Institute, Hamilton, Canada; Department of Health Research Methodology, Evidence & Impact, McMaster University, Hamilton, Canada; Division of Nephrology, Department of Medicine, McMaster University, Hamilton, Canada; Department of Health Research Methodology, Evidence & Impact, McMaster University, Hamilton, Canada; Department of Renal Medicine, St George Hospital, Kogarah, Australia; NHMRC Clinical Trials Centre, University of Sydney, Camperdown, Australia; MRC Population Health Research Unit, Clinical Trial Service Unit and Epidemiological Studies Unit, Nuffield Department of Population Health, University of Oxford, Oxford, UK; NHMRC Clinical Trials Centre, University of Sydney, Camperdown, Australia; Department of Renal Medicine, Concord Repatriation General Hospital, Sydney, Australia; School of Cardiovascular and Metabolic Health, University of Glasgow, Glasgow, UK; Department of Nephrology, St George Hospital, Sydney, NSW, Australia; Faculty of Medicine & Health, UNSW, Sydney, NSW, Australia; Renal and Metabolic Division, The George Institute for Global Health, Sydney, NSW, Australia; Centre d'Investigations Cliniques-Plurithématique 14-33, Université de Lorraine, Inserm U1116, CHRU Nancy, and F-CRIN INI-CRCT (Cardiovascular and Renal Clinical Trialists), Nancy, France; Medicine and Nephrology-Dialysis Departments, Princess Grace Hospital, and Monaco Private Hemodialysis Centre, Monaco, Monaco; Renal-Electrolyte and Hypertension Division and Center for Clinical Epidemiology and Biostatistics, Perelman School of Medicine, University of Pennsylvania, Philadelphia, PA, USA; Department of Clinical Research and Epidemiology, DZHI and University Hospital, Würzburg, Germany; Canadian Vigour Center, Edmonton, Canada; Division of Cardiology, Department of Medicine, University of Alberta, Edmonton, Canada; Population Health Research Institute, Hamilton, Canada; Department of Health Research Methodology, Evidence & Impact, McMaster University, Hamilton, Canada; Divisions of Cardiology and Perioperative Medicine, Department of Medicine, McMaster University, Hamilton, Canada; Division of Nephrology, Department of Medicine, Faculty of Medicine, Memorial University Newfoundland, St. John's, Canada; Department of Nephrology, University Medical Center Groningen, University of Groningen, Groningen, The Netherlands; Division of Nephrology, Department of Medicine, University of Alberta, Edmonton, Canada; Population Health Research Institute, Hamilton, Canada; Department of Health Research Methodology, Evidence & Impact, McMaster University, Hamilton, Canada

**Keywords:** dialysis, event diagnostic criteria, heart failure, meta-epidemiology

## Abstract

**Background and hypothesis:**

Heart failure is characterized as cardiac dysfunction resulting in elevated cardiac filling pressures with symptoms and signs of congestion. Distinguishing heart failure from other causes of similar presentations in patients with kidney failure is challenging but necessary, and is needed in randomized controlled trials (RCTs) to accurately estimate treatment effects. The objective of this study was to review heart failure events, their diagnostic criteria, and adjudication in RCTs of patients with kidney failure treated with dialysis. We hypothesized that heart failure events, diagnostic criteria, and adjudication were infrequently reported in RCTs in dialysis.

**Methods:**

We conducted a meta-epidemiologic systematic review of RCTs from high-impact medical, nephrology, and cardiology journals from 2000 to 2020. RCTs were eligible if they enrolled adults receiving maintenance dialysis for kidney failure and evaluated any intervention.

**Results:**

Of 561 RCTs in patients receiving dialysis, 36 (6.4%) reported heart failure events as primary (10, 27.8%) or secondary (31, 86.1%) outcomes. Ten of the 36 (27.8%) RCTs provided heart failure event diagnostic criteria and five of these (50%) adjudicated heart failure events. These 10 RCTs included event diagnostic criteria for heart failure or heart failure hospitalizations, and their criteria included dyspnoea (5/10), oedema (2/10), rales/crackles (4/10), chest X-ray pulmonary oedema or vascular redistribution (4/10), treatment in an acute setting (6/10), and ultrafiltration or dialysis (4/10). No study explicitly distinguished heart failure from volume overload secondary to non-adherence or underdialysis.

**Conclusion:**

Overall, we found that heart failure events are infrequently reported in RCTs in dialysis and are heterogeneously defined. Further research is required to develop standardized diagnostic criteria that are practical and meaningful to patients and clinicians.

## Graphical Abstract

**Graphical Abstract:**
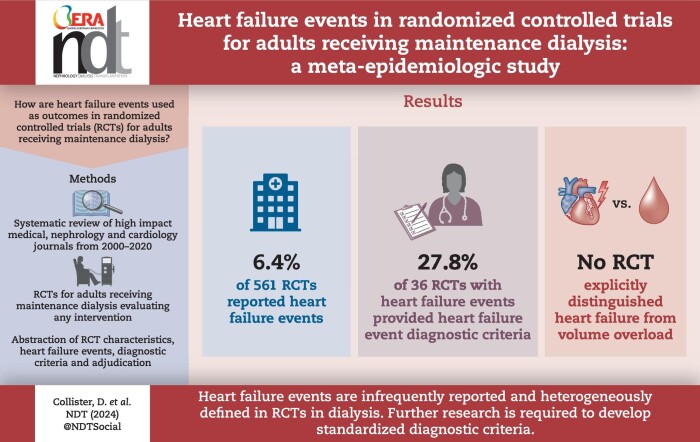

KEY LEARNING POINTS **What was known:**

- Heart failure is common in patients with kidney failure treated with dialysis and must be properly distinguished from other causes of shortness of breath. Distinguishing heart failure is necessary to accurately estimate treatment effects in clinical trials of treatments for heart and vascular disease in patients receiving dialysis.

**This study adds:**

- In this review of 561 clinical trials in dialysis, we found that of the 36 studies (6.4%) that reported heart failure events as study outcomes, only 10/36 (27.8%) provided heart failure event diagnostic criteria and these criteria varied from study to study.

**Potential impact:**

- Further research is needed to develop standardized event definitions for heart failure in dialysis so that treatment effects can be accurately accessed and communicated to knowledge users.

## INTRODUCTION

Heart failure is a clinical syndrome with symptoms and/or signs caused by a structural and/or functional cardiac abnormality corroborated by objective evidence of congestion and/or elevated ventricular filling pressures [1]. Heart failure is a common comorbidity in patients with kidney failure receiving dialysis and is an important contributor to morbidity and mortality [2, 3]. While cardiovascular (CV) events are clearly recognized as important to patients with kidney disease, there is uncertainty as to whether CV events, including heart failure, are defined adequately in patients with kidney failure [4–6]. Heart failure events are particularly problematic because they share many features of pure volume overload due to dietary sodium and fluid non-adherence or underdialysis that can make the entities frequently clinically indistinguishable in clinical and research settings.

In randomized controlled trials (RCTs), outcome events are ideally important to both patients and clinicians by virtue of their prognostic relevance (e.g. impact on survival, function, or quality of life). Outcome events should also be easily differentiated from other events that are dissimilar in their importance and pathophysiology. We make a distinction between a diagnostic definition, which provides the concept of a diagnosis (e.g. elevated cardiac filling pressures due to cardiac dysfunction), and diagnostic criteria, which provide explicit criteria that clinicians can use to establish a diagnosis (e.g. diagnostic criteria for heart failure requires dyspnoea and at least one of the of the following signs [i.e. elevated jugular venous pressure (JVP), respiratory crackles, S3] and either radiographic findings [i.e. vascular redistribution, interstitial pulmonary oedema] with implementation of heart failure treatments such as diuretics or ultrafiltration and documented clinical improvement). When diagnostic criteria are poorly specified, they introduce ‘noise’, which quickly diminishes statistical power and makes the interpretation of treatment effects challenging. In heart failure populations without concomitant kidney failure, standardized consensus diagnostic criteria for heart failure events exist [7] and these diagnostic criteria help ensure events are of similar meaning to stakeholders (i.e. patients, caregivers, healthcare providers, researchers, industry, regulators), are prognostically relevant, comparable across clinical studies, and ideally have shared pathophysiology to facilitate the identification of effective therapies.

Volume overload due to dietary salt and water excess or inadequate fluid removal with dialysis rather than primary cardiac dysfunction can appear identical to heart failure in dialysis and have similar associations with morbidity and mortality. However, imagine an intervention that acts purely by improving cardiac structure and function. While this would reduce the risk of episodes of heart failure, it may have no effect on episodes of pure volume overload due to dietary non-adherence or inadequate fluid removal with dialysis. In a population where 50% of the events that look like heart failure are due to actual heart failure and 50% are due to volume overload, the effect of the treatment, which is only on actual heart failure, would be cut in half if the two types of event cannot be reliably distinguished. For an intervention that works purely on volume overload and not on heart failure, the same issue but in reverse applies. The situation is even more difficult because the pathophysiology of heart failure and volume overload are complex and invariably intertwined and interventions could act, to varying degrees, on both clinical syndromes [8–10]. The issue of differentiating heart failure events from volume overload is not new to nephrology and RCTs have had to consider this issue for many years [11]. To help consider the current state of heart failure events, and their diagnostic criteria and shape future definitions in dialysis, we systematically reviewed heart failure events, and how they are defined in RCTs of patients receiving maintenance dialysis.

## MATERIALS AND METHODS

### Objectives

To systematically review the proportion of dialysis RCTs where heart failure events (heart failure/acute decompensated heart failure, heart failure emergency room visit, or hospitalization, heart failure death) are reported as a primary or secondary outcomes, examine differences in the design and conduct of RCTs with and without heart failure events as outcomes, and explore heart failure diagnostic criteria and adjudication.

### Design

A meta-epidemiologic study of RCTs in adults with kidney failure (haemodialysis, peritoneal dialysis) receiving maintenance dialysis published in high-impact general medical, nephrology, and cardiology journals indexed in PubMed.

### Study selection

PubMed was searched to identify RCTs published in high-impact general medical journals including the *Annals of Internal Medicine*, the *British Medical Journal*, the *Journal of the American Medical Association* (JAMA), *the Lancet*, the *New England Journal of Medicine*, and high-impact nephrology journals including the *American Journal of Kidney Diseases*, the *Clinical Journal of the American Society of Nephrology* (CJASN), the *Journal of the American Society of Nephrology* (JASN), *Kidney International*, and *Nephrology Dialysis Transplantation*. We also included high-impact cardiology journals including *Circulation*, the *Journal of the American College of Cardiology* (JACC), and the *European Heart Journal*. We used the search term ‘randomized controlled trial’ limiting inclusion to publication dates from 1 January 2001 until 31 December 2020.

A purposive sample was drawn by including RCTs of any sample size, design (e.g. parallel, crossover, cluster, stepped wedge) and published between January 1, 2001 until December 31, 2020 in any language that met the following eligibility criteria:

- included only participants at least 18 years of age;
- included patients with kidney failure treated with either maintenance dialysis but not kidney transplantation or acute kidney replacement therapy;
- was not a secondary analysis or substudy;
- was not a long-term follow-up or extension study; and
- did not have more than one intervention or sequential stages, to avoid the possibility of duplicate data or incomplete reporting.

Studies were uploaded in Covidence. Two reviewers (L.P., A.B.) independently reviewed all abstracts and full texts for eligibility. All disagreements were resolved by a third reviewer (D.C.).

### Data abstraction

Two reviewers (L.P., A.B.) independently extracted data from each study using the primary report, supplementary material, protocol, and registration if necessary. Abstracted data elements included the study author, journal, design (parallel, crossover, other) population (haemodialysis, peritoneal dialysis, both), intervention, comparator (placebo, usual care, active), blinding (blinded, open-label, unclear), country, sample size, follow-up duration, whether the primary outcome was statistically significant defined by a *P* value <0.05 (yes, no, unclear), and funding (non-industry, industry, both, unclear). We then focused on RCTs with heart failure events as primary, secondary, or composite outcomes. The following data elements were abstracted for heart failure events as primary, secondary, or composite outcomes (e.g. heart failure death with other nonfatal CV events or a hospitalization for heart failure with other fatal or nonfatal CV events or mortality), event rates, diagnostic criteria including symptoms (dyspnoea at rest, dyspnoea on exertion, orthopnea, paroxysmal nocturnal dyspnoea, oedema), and New York Heart Association stage, signs (oedema, elevated jugular venous pulse, crackles/rales, the presence of a S3), imaging abnormalities (chest X-ray, ultrasound echocardiography, radionuclide imaging, left heart catheterization), cardiac biomarker abnormalities (brain natriuretic peptide, N-terminal prohormone brain natriuretic peptide), planned or unplanned healthcare utilization (clinic, dialysis unit, emergency room, hospital), the need for dialysis/ultrafiltration, any additional treatments (e.g. increase in oral diuretics, intravenous diuretics, inotropes, non-invasive mechanical ventilation, mechanical ventilation, mechanical circulatory support), whether an alternative diagnosis could be present, and whether adjudication was performed. We did not include any tertiary outcomes or safety outcomes that were not primary or secondary outcomes including individual adverse events that may be related to heart failure (e.g. dyspnoea, oedema, and fatigue). We did not consider the source of data (e.g. case report forms, supporting documents, linkages with registries/administrative databases). All disagreements were resolved by a third reviewer (D.C.).

### Statistical analysis

Descriptive statistics included frequency and the percentage, mean, and standard deviation, or medians and first and third quartiles (Q1: Q3). RCTs with heart failure events were compared to RCTs without heart failure events with regards to design, populations, interventions, comparators, and outcomes using parametric and non-parametric statistical tests. The level of statistical significance was *P* < .05. All analyses were performed using Stata (StataCorp. 2015. Stata Statistical Software: Release 14. College Station, TX, USA: StataCorp LP).

## RESULTS

**Figure 1:**
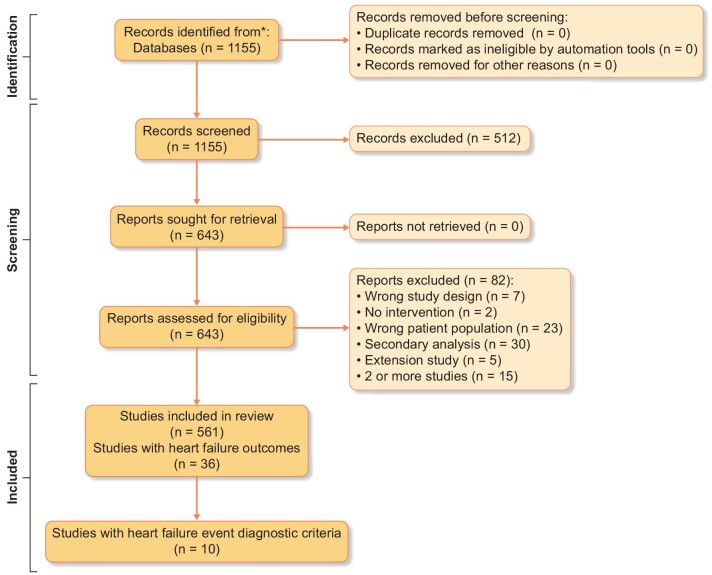
PRISMA flow diagram.

Of 1155 potentially eligible manuscripts, 561 RCTs were included (Figure 1), and their characteristics are shown in Table 1. Of these 561 RCTs, 391 (69.7%) were parallel RCTs, 452 (80.6%) were in haemodialysis from Europe (217, 38.6%) and North America (166, 29.5%), the median size was 60 participants (Q1: Q3 26:151) and the median duration of follow-up was 154 days (Q1: Q3 42:365). Many different classes of interventions were evaluated and compared to usual care (135, 24.1%), placebo (136, 24.3%), or an active therapy (289, 51.6%) in blinded (211, 37.6%) or open-label (222, 39.6%) administrations with approximately half of RCTs having statistically significant (*P* < .05) primary results. RCTs were funded by non-industry (200, 35.7%), industry (190, 33.9%), or both (45, 8.0%).

**Table 1:** Characteristics of included studies.

		All trials(*N* = 561)	Trial with heart failureevents(*N* = 36)	Trials without heart failureevents(*N* = 525)	*P* value^a^
Design	Parallel	391 (69.7%)	35 (97.2%)	356 (67.8%)	<.001
	Crossover	157 (28.0%)	1 (2.8%)	156 (29.7%)	
	Other	13 (2.3%)	0 (0%)	13 (2.5%)	
Comparator	Usual care	135 (24.1%)	2 (5.6%)	125 (23.9%)	.049
	Placebo	136 (24.3%)	12 (33.3%)	122 (23.3%)	
	Active	289 (51.6%)	22 (61.1%)	277 (52.9%)	
Blinding	Blinded	211 (37.6%)	15 (41.7%)	196 (37.3%)	.078
	Open-label	222(39.6%)	18 (50.0%)	204 (38.9%)	
	Unclear	128 (22.8%)	3 (8.3%)	125 (23.8%)	
Size	Median (IQR)	60 (26, 151)	321 (129, 747)	60 (25, 140)	<.001
Population	Haemodialysis	452 (80.6%)	30 (83.3%)	422 (80.4%)	1.000
	Peritoneal dialysis	81 (14.4%)	5 (13.9%)	76 (14.5%)	
	Both	28 (5.0%)	1 (2.8%)	27 (5.1%)	
Region	Intercontinental	40 (7.1%)	5 (13.9%)	35 (6.7%)	.153
	Asia	107 (19.0%)	10 (27.7%)	96 (18.3%)	
	Europe	217 (38.6%)	15 (41.7%)	203 (38.7%)	
	North America	166 (29.5%)	6 (16.7%)	160 (30.5%)	
	South America	9 (1.6%)	0 (0%)	9 (1.7%)	
	Oceania	21 (3.7%)	0 (0%)	21 (4.0%)	
Intervention	Anaemia	58 (10.3%)	2 (5.6%)	56 (10.7%)	<.001
	Antibiotics	8 (1.4%)	0 (0%)	8 (1.5%)	
	Anticoagulation	22 (3.9%)	1 (2.8%)	21 (4.0%)	
	Bone mineral disorder	76 (13.6%)	5 (13.9%)	71 (13.5%)	
	Cardiovascular	62 (11.1%)	14 (39.9%)	48 (9.1%)	
	Exercise	16 (2.9%)	1 (2.8%)	15 (2.9%)	
	Haemodialysis	123 (21.9%)	6 (16.7%)	117 (22.3%)	
	Peritoneal dialysis	44 (7.8%)	4 (11.1%)	40 (7.6%)	
	Symptoms	25 (4.5%)	0 (0%)	25 (4.8%)	
	Other	83 (14.8%)	3 (8.3%)	80 (15.2%)	
	Vascular access	44 (7.8%)	0 (0%)	44 (8.4%)	
Follow-up	Median (IQR)	154 days(42–365)	682 days(365–919)	121 days(42–365)	<.001
Primary outcome	Significant (*P* < .05)	281 (50.1%)	9 (25.0%)	269 (51.2%)	.001
	Non-significant (*P* ≥ .05)	185 (33.0%)	27 (75.0%)	163 (31.1%)	
	Unclear	95 (16.9%)	0 (0%)	93 (17.7%)	
Funding	Non-industry	200 (35.7%)	17 (47.2%)	183 (34.9%)	.107
	Industry	190 (33.9%)	12 (33.3%)	178 (33.9%)	
	Both	45 (8.0%)	4 (11.1%)	41 (7.8%)	
	Unclear	126 (22.5%)	3 (8.3%)	123 (23.4%)	

Note: SD = standard deviation, IQR = interquartile range, N/A = not applicable

^a^Comparison between trials with and without heart failure outcomes using a *t*-test, Fisher's exact test as appropriate was adjusted for each journal but not shown.

Of the 561 RCTs, 36 (6.4%) reported heart failure events as either primary (10, 27.8%) or secondary outcomes (31, 86.1%) (Table 2) consisting of (35, 97.2%) parallel RCTs in haemodialysis (30, 83.3%) from Europe (15, 41.7%), Asia (10, 27.7%), and North America (6, 16.7%) with a mean size of 321 participants (Q1: Q3 129, 747) and duration of follow-up 682 days (Q1: Q3 365, 919). Interventions were compared to active therapies (22, 61.1%) or placebo (12, 33.3%) in open-label (18, 50.0%) or blinded (15, 41.7%) administrations with only a quarter of RCTs having statistically significant (*P* < .05) primary results. RCTs were funded by non-industry (17, 47.2%), industry (12, 33.3%), or both (4, 11.1%). Sixteen of 36 (44.4%) of RCTs had a heart failure event as part of a primary or secondary composite outcome that typically included a heart failure hospitalization or heart failure death with other fatal or nonfatal CV events. Compared to RCTs without heart failure events as outcomes, RCTs with heart failure events as outcomes were more likely to be parallel in design and larger with shorter follow-up but were less likely to have statistically significant results in their primary outcomes (Table 1).

**Table 2:** Heart failure outcomes.

		All trials (*N* = 561)	Trial with heart failure events (*N* = 36)
Heart failure outcomes	PrimaryOutcome (individually or as a composite)	10 (1.8%)^a^	10 (27.7%)
	Heart failure is part of a primary composite outcome	8 (1.4%)	8 (22.2%)
	Secondary outcome (individually or as a composite)	31 (5.5%)^b^	31 (86.1%)
	Heart failure is part of a secondary composite outcome	9 (1.6%)	9 (25.0%)
	Heart failure is part of a primary or secondary composite outcome	16 (2.9%)	16 (44.4%)

a Six hospitalization for heart failure, two heart failure death, one both, one unclear

b Six hospitalization for heart failure, nine heart failure death, four both, two unclear, seven fluid overload, one heart failure death or death due to pulmonary oedema secondary to exogenous fluid, one heart failure death and unclear, one *de novo* heart failure without hospitalization

Among the 36 RCTs that reported heart failure events, 10 (27.8%) reported diagnostic criteria for heart failure and were predominantly available only in protocols and/or supplementary material. The characteristics of these 10 RCTs are shown in Table 3. Eight of 10 (80%) of these RCTs with diagnostic criteria for heart failure had a heart failure event as a part of their primary composite endpoint. All 10 RCTs were in haemodialysis and interventions included renin angiotensin system inhibitors (four, 40%), mineralocorticoid receptor antagonists (two, 20%), parathyroid hormone receptor antagonists (one, 10%), intravenous iron (one, 10%), erythropoietin stimulating agents to achieve different haemoglobin targets (one, 10%), and vitamin D analogues (one, 10%). Eight of 10 (80%) included heart failure in their primary outcome, typically within a composite of fatal or nonfatal CV events or hospitalization for CV events and 5 of 10 (50%) had event adjudication. The frequency of heart failure hospitalizations was reported in 9 of 10 (90%) RCTs and heart failure hospitalization event rates per patient year(s) of follow-up were estimated on the number of events and mean/median follow-up times in 5 of 10 (50%) studies and varied from 0.82–0.85/100 patient years to 11.48–18.62/100 patient years.

**Table 3:** Characteristics of included studies with heart failure event definitions (10 studies).

Author	Year	Population(*n*)	Intervention	Comparator	Duration	Primary outcome	Does primary outcome include a heart failure event?	Adjudication	Heart failureevent rate^a^active (%)comparator (%)
Chertow [37]	2012	HD(3883)	Cinacalcet	Placebo	median 21.1/17.5 months study drug exposure	Time to death or first nonfatal cardiovascular event	Yes	Yes	206/1948 (10.6%)6.01/100 PY236/1935 (12.2%)8.36/100 PY
Cice [38]	2010	HD(332)	Telmisartan	Placebo	mean 35.5 (SD 8.5) months,median 36 months	(i) All-cause mortality(ii) Cardiovascular death(iii) Hospital admission for management of worsening congestive heart failure	Yes	No	56/165 (33.9%)11.48/100 PY92/167 (55.1%)18.62/100 PY
MacDougall [17]	2019	HD(2141)	High dose IV iron, proactive	Low dose IV iron, reactive	median 2.1 years	Nonfatal MI, nonfatal stroke, hospitalization for heart failure, death from any cause	Yes	Yes	51/1093 (4.7%)2.22/100 PY70/1048 (6.7%)3.18/100 PY
Matmsumoto [39]	2014	HD(309)	Spironolactone	Control	3 years	Death from cardiovascular events or hospitalization for cardiovascular events	Yes	No	1/147 (0.7%)3/152 (2.0%)
Parfrey [40]	2005	HD(596)	Haemoglobin target13.5–14.5 g/dl	Haemoglobin target9.5–11.5 g/dl	96 weeks	Left ventricular volume index	No	No	11/296 (3.7%)12/300 (4.0%)(efficacy)11/296 (3.7%)19/300 (6.3%)(adverse event)
Shoji [16]	2018	HD(1289)	Oral alfacalcidiol	Control	mean 3.6 yearsmedian 4.0 years	Fatal and nonfatal cardiovascular events, coronary intervention of bypass grafting, lower limb artery intervention, or bypass grafting	Yes	Yes	15/488 (3.1%)0.85/100 PY14/476 (2.9%)0.82/100 PY
Suzuki [41]	2008	HD(366)	Angiotensinogen receptor blocker	Control	36 months	Fatal and nonfatal cardiovascular events	Yes	No	14/180 (7.8%)29/180 (16.1%)
Takahashi [42]	2006	HD(80)	Candesartan	Control	mean 19.4 (SD 1.2) months	Cardiovascular events	Yes	Yes	5/43 (11.6%)7.19/100 PY11/37 (29.7%)18.39/100 PY
Walsh [15]	2015	HD(154)	Eplerenone	Placebo	13 weeks	Permanent discontinuation of study drug due to hyperkalaemia or significant hypotension	No	No	Not reported
Zannad [43]	2006	HD(397)	Fosinopril	Placebo	24 months	Cardiovascular events	Yes	Yes	54/397 (13.6%)(pooled)

^a^Heart failure event rate calculated if mean/median follow-up reported

The diagnostic criteria for heart failure events in the 10 RCTs that included heart failure diagnostic criteria are shown in Table 4. Heart failure events with definitions included heart failure (three, 30%), heart failure hospitalization (five, 50%) and a composite of heart failure hospitalization and death due to heart failure/pump failure (two, 20%). Heart failure diagnostic criteria differed across RCTs with regards symptoms, signs, laboratory tests, imaging, healthcare utilization, and interventions (Table 5). Across the 10 RCTs with heart failure diagnostic criteria, symptoms were required in five RCTs (50%) including at least one of dyspnoea (five, 50%), orthopnoea (one, 10%), paroxysmal nocturnal dyspnoea (one, 10%), or fatigue (two, 20%), and signs were required in five RCTs (50%) including peripheral oedema (two, 20%), rales/crackles (four, 40%), an S3 (two, 20%), or elevated JVP (four, 40%). Two RCTs (20%) required an increase in interdialytic weight gain and one (10%) required an elevated brain natriuretic peptide as part of diagnostic criteria. Imaging showing pulmonary venous congestion was required in diagnostic criteria including interstitial oedema (four, 40%) and vascular redistribution (four, 40%) or an elevated left ventricular end diastolic pressure/pulmonary wedge capillary pressure (three, 30%) or other findings on echocardiography (two, 20%). For 6 of the 10 RCTs (60%) the heart failure outcome required presentation to acute care setting (e.g. dialysis unit, emergency room, hospitalization) of which three out of six (50%) of these specified that it had to be unplanned and three out of six (50%) the primary reason for hospitalization. Intensification of medical therapy was defined as one or more of the following in these RCTs: 4 out of 10 (40%) RCTs required ultrafiltration or dialysis, 1 out of 10 (10%) RCTs required intravenous diuretics, and 1 out of 10 (10%) RCTs required a mechanical or surgical intervention. No trial distinguished heart failure from volume overload (e.g. from non-adherence with dietary sodium/fluid restriction or dialysis) and only one trial permitted heart failure to occur during an ongoing hospitalization for another condition in which heart failure became a major component of the hospitalization.

**Table 4:** Heart failure event diagnostic criteria.

Author	Event	Diagnostic criteria
Chertow [37](2012)	Heart failure hospitalization	An unplanned presentation to an acute care setting (hospital or dialysis unit) with signs/symptoms of volume overload (see below) and the patient received mechanical fluid removal therapy (e.g. ultrafiltration or dialysis) OR acute exacerbation of HF with symptomatic pulmonary oedema during an ongoing hospitalization for another condition in which HF becomes a major component of the hospitalization provided that the patient received a mechanical fluid removal (e.g. ultrafiltration or dialysis).
Cice [38](2010)	Pump failure death	Pump failure death was defined as death due to progressive deterioration of heart failure, acute pulmonaryoedema, or cardiogenic shock.
Cice [38](2010)	Heart failure hospitalization	A CHF hospital admission was defined as admission to hospital necessitated by heart failure and primarily for its treatment or when heart failure was a major component of the hospital admission of the patient. A patient admitted to a hospital for CHF decompensation had to have documented signs and symptoms of worsening heart failure requiring intravenous drug administration and a supplementary haemodialysis treatment.
MacDougall [17](2019)	Death due to heart failure	Death due to heart failure refers to a death occurring in the context of new or worsening clinical manifestations of heart failure (see ‘hospitalization for heart failure definition’—section 5.2.6, below) without evidence of another cause of death (e.g. acute myocardial infarction). In general, the new or worsening clinical manifestations should require the initiation of, or an increase in, treatment directed at heart failure, or occur in a patient already receiving maximal therapy for heart failure. However, if time does not allow for the initiation of, or an increase in, treatment directed at heart failure or if the circumstances were such that doing so would have been inappropriate (e.g. patient refusal), the EPAC will adjudicate based on clinical presentation and, if available, investigative evidence.
MacDougall [17](2019)	Heart failure hospitalization	For the diagnosis of hospitalization for heart failure, there should be emergency/unplanned admission to a hospital setting (emergency room, observation, or inpatient unit) that results in at least one overnight stay (i.e. a date change) with fulfilment of the following criteria:There should be:(i) Clinical manifestations of new or worsening heart failure including at least one of the following:• New or worsening dyspnoea on exertion• New or worsening dyspnoea at rest• New or worsening fatigue/decreased exercise tolerance• New or worsening orthopnoea• New or worsening PND• New or worsening lower limb or sacral oedema• New or worsening pulmonary crackles/crepitations• New or worsening elevation of JVP• New or worsening third heart sound or gallop rhythmAnd(ii) Investigative evidence of structural or functional heart disease (if available) with at least oneof the following:• Radiological evidence of pulmonary oedema/congestion or cardiomegaly.• Imaging (e.g. echocardiography, cardiac magnetic resonance imaging, radionuclideventriculography) evidence of an abnormality (e.g. left ventricular systolic dysfunction,significant valvular heart disease, left ventricular hypertrophy).• Elevation of B-type natriuretic peptide (BNP) or NT-proBNP levels.• Other investigative evidence of structural or functional heart disease (e.g. evidence obtainedfrom pulmonary artery catheterization).And(iii) Need for new/increased therapy specifically for the treatment of heart failure including atleast one of the following:• Initiation of intravenous diuretic, inotrope, vasodilator, or other recognized intravenous heartfailure treatment or up titration of such intravenous therapy if already receiving it.• Mechanical or surgical intervention (e.g. mechanical or non-invasive ventilation, mechanicalcirculatory support).• Alteration to the dialysis schedule to facilitate extra mechanical fluid removal^a^ (this may include extra dialysis sessions or longer dialysis).And(iv) The EPAC should be satisfied that heart failure was the primary disease process accounting for the clinical presentation.
Matmsumoto [39](2014)	Heart failure hospitalization	New occurrence or exacerbation of heart failure that was not improved by water removal through dialysis (clinical symptoms together with left ventricular dysfunction by echocardiography according to the American Heart Association/ACC guidelines).
Parfrey [40](2005)	Heart failure	*De novo* heart failure was defined as dyspnoea at rest with two of the following: increased JVP, bilateral basal crackles, radiographic pulmonary hypertension, and radiographic interstitial oedema.
Shoji [16](2018)	Heart failure hospitalization	Congestive heart failure (NYHA grade III or IV) requiring hospitalization, excluding dyspnoea due to non-cardiac causes.
Suzuki [41](2008)	Heart failure	Congestive heart failure was defined according to the guidelines of the ACC and AHA (Hunt SA, Baker DW, Chin MH, *et al. Circulation* 104:2996–3007, 2001).
Takahashi [42](2006)	Heart failure hospitalization	Congestive heart failure requiring hospitalization (New York Heart Association class III or IV).
Walsh [15](2015)	Heart failure	The definition of congestive heart failure requires at least one of the following clinical signs (i.e. any of the following signs: elevated JVP, respiratory rales/crackles, crepitations, or presence of S3) and at least one of the following radiographic findings (i.e. vascular redistribution, interstitial pulmonary oedema, or frank alveolar pulmonary oedema).
Zannad [43](2006)	Heart failure hospitalization	Heart failure was defined by a period of >24 h hospitalization for new onset or worsening of dyspnoea with signs and symptoms of clinical and/or radiological signs of peripheral and/or pulmonary congestion and documented worsening of cardiac function (increase in X-ray cardiothoracic ratio or increase of echocardiogram LV dimension with decrease in LV shortening fraction or increase in heart catheterization pulmonary capillary wedge pressure or LV filling pressure). In addition or alternatively, the dialysis strategy had to be changed for up to one consecutive month, including increase in weekly dialysis duration >20% and/or increase in baseline weight >1 kg, and/or switch to hemofiltration. These changes could not be in response to an omission or reduction in routine dialysis.

Note: uniform definitions for CV outcomes and heart failure developed in 2014 and 2017 by the SCTI/FDA

Diagnostic criteria were abstracted the primary publication, protocol, or supplementary material.

^a^When classifying an event as meeting the definition of ‘hospitalization for heart failure’, the adjudication committee will record whether or not the treatment administered included extra mechanical fluid removal.

**Table 5:** Heart failure and heart failure hospitalization diagnostic criteria components across RCTs^a^.

	Symptoms				Signs				Weight	Laboratory	Imaging				Healthcare utilization			Intervention		
	SOB	Orthopnoea	PND	Fatigue	Peripheral oedema	Rales/crackles	S3	JVP	IDWG	BNP	CXR oedema	CXR vascular redistribution	LVEDP PCWP	Echo	Acute	Not planned	Primary reason	UF/dialysis	IV diuretics	Mechanical/surgical
Chertow [37]	1	0	0	0	0	1	0	1	0	0	1	1	1	0	1	1	0	1	0	0
Cice[38]	0	0	0	0	0	0	0	0	0	0	0	0	0	1	1	1	1	1	0	0
MacDougall [17]	1	1	1	1	1	1	1	1	0	1	1	1	1	0	1	1	1	1	1	1
Matsumoto [39]	0	0	0	0	0	0	0	0	0	0	0	0	0	0	1	0	0	0	0	0
Parfrey [40]	1	0	0	0	0	1	0	1	1	0	1	1	0	1	0	0	0	0	0	0
Shoji[16]	1	0	0	1	0	0	0	0	0	0	0	0	0	0	1	0	1	0	0	0
Suzuki [41]	0	0	0	0	0	0	0	0	0	0	0	0	0	0	0	0	0	0	0	0
Takahashi [42]	0	0	0	0	0	0	0	0	0	0	0	0	0	0	0	0	0	0	0	0
Walsh[15]	0	0	0	0	0	1	1	1	1	0	1	1	0	0	0	0	0	0	0	0
Zannad [43]	1	0	0	0	1	0	0	0	0	0	0	0	1	0	1	0	0	1	0	0
Total	5/10(50%)	1/10(10%)	1/10(10%)	2/10(20%)	2/10(20%)	4/10(40%)	2/10(20%)	4/10(40%)	2/10(20%)	1/10(10%)	4/10(40%)	4/10(40%)	3/10(30%)	2/10(20%)	6/10(60%)	3/10(30%)	3/10(30%)	4/10(40%)	1/10(10%)	1/10(10%)

Note: SOB = shortness of breath, PND = paroxysmal nocturnal dyspnoea, JVP = jugular venous pressure, IDWG = interdialytic weight gain, BNP = brain natriuretic peptide, CXR = chest X-ray, LVEDP = left ventricular end diastolic pressure, PCWP = pulmonary capillary wedge pressure, echo = echocardiogram, UF = ultrafiltration, IV = intravenous

^a^Excludes heart/pump failure deaths

## DISCUSSION

### Key findings

In this meta-epidemiologic systematic review of 561 RCTs in adults with kidney failure requiring maintenance dialysis, ∼1 in 20 RCTs had heart failure events as primary or secondary outcomes of which one in three included the heart failure event as a component of a composite outcome with other non-heart failure CV events. Only 10 RCTs had heart failure diagnostic criteria, all of which were applied to patients receiving haemodialysis and there was heterogeneity across criteria relative to symptoms, signs, laboratory tests, imaging, health care utilization, and interventions without any differentiation from volume overload due to non-adherence or underdialysis.

### Heart failure reporting in other settings

The American College of Cardiology (ACC) and the American Heart Association (AHA) have published clinical data standards for clinical care and research that include harmonized and standardized diagnostic criteria of key data elements including symptoms, signs, and diagnostic procedures but not outcomes similar to the Clinical Data Interchange Standards Consortium [12, 13]. There are uniform definitions for CV outcomes and heart failure events developed in 2014 and 2017 by the Standardized Data Collection for Cardiovascular Trials Initiative (SCTI) and the US Food and Drug Administration (FDA) following the lack of uniform definitions for key endpoint events and need for objective criteria and uniform reporting [7]. Standardized diagnostic criteria for the evaluation of heart failure therapies were also published in 2020 by the Heart Failure Collaboratory Academic Research Consortium [14] (Table 6). Of note, only three out of 10 (30%) of dialysis RCTs with heart failure diagnostic criteria included in this study's sample were published after 2014 [15–17]. A heart failure hospitalization typically includes an unscheduled hospital admission for a primary diagnosis of heart failure with typical symptoms, signs, and diagnosis testing results consistent with the diagnosis of heart failure including elevated brain natriuretic peptide, radiological evidence of congestion, and either echocardiographic or invasive evidence of elevated filling pressure. The patient should also receive treatment specifically directed at heart failure including at least one of the following: significant augmentation in oral diuretic therapy, initiation of intravenous diuretic or vasoactive agent, or mechanical circulatory support of fluid removal (including ultrafiltration, hemofiltration, dialysis). These definitions do not specify how these conditions are differentiated from usual care in patients receiving maintenance dialysis where the symptoms and signs are often chronically present, due to conditions other than heart failure, and when maximal or near maximal therapy is always being received and is not specific for heart failure.

**Table 6:** Heart failure hospitalization definitions.

Source	Heart failure hospitalization definition
SCTI and the United States FDA (2017)	**A Heart Failure Hospitalization** is defined as an event that meets **ALL** of the following criteria:1) The patient is admitted to the hospital with a ***primary diagnosis*** of HF2) The patient's length of stay in hospital extends for **at least 24 hours** (or a change in calendar date if the hospital admission and discharge times are unavailable)3) The patient exhibits documented new or worsening symptoms due to HF on presentation, including **at least ONE** of the following:a. Dyspnoea (dyspnoea with exertion, dyspnoea at rest, orthopnea, PND)b. Decreased exercise tolerancec. Fatigued. Other symptoms of worsened end-organ perfusion or volume overload (must be specified and described by the protocol)4) The patient has objective evidence of new or worsening HF, consisting of **at least TWO** physical examination findings **OR** one physical examination finding and **at least ONE** laboratory criterion, including:a. Physical examination findings considered to be due to heart failure, including new or worsened:i. Peripheral oedemaii. Increasing abdominal distention or ascites (in the absence of primary hepatic disease)iii. Pulmonary rales/crackles/crepitationsiv. Increased JVP and/or hepatojugular refluxv. S_3_ gallopvi. Clinically significant or rapid weight gain thought to be related to fluid retentionb. Laboratory evidence of new or worsening HF, if obtained within 24 hours of presentation, including:i. Increased BNP/N-terminal pro-BNP (NT-proBNP) concentrations consistent with decompensation of heart failure (such as BNP >500 pg/ml or NT-proBNP >2000 pg/ml). In patients with chronically elevated natriuretic peptides, a significant increase should be noted above baseline.ii. Radiological evidence of pulmonary congestioniii. Non-invasive diagnostic evidence of clinically significant elevated left- or right-sided ventricular filling pressure or low cardiac output. For example, echocardiographic criteria could include: septal or lateral E/e’ >15 or >12, respectively; D-dominant pulmonary venous inflow pattern; plethoric inferior vena cava with minimal collapse on inspiration; or decreased left ventricular outflow tract minute stroke distance (time velocity integral)**OR**iv. Invasive diagnostic evidence with right heart catheterization showing a pulmonary capillary wedge pressure (pulmonary artery occlusion pressure) ≥18 mmHg, central venous pressure ≥12 mmHg, or a cardiac index <2.2 l/min/m^2^**Note: All results from diagnostic tests should be reported, if available, even if they do not meet the above criteria, because they provide important information for the adjudication of these events.**5) The patient receives **at least ONE** of the following treatments specifically for HF:a. Significant augmentation in oral diuretic therapy (e.g. doubling of loop diuretic dose, initiation of maintenance loop diuretic therapy, initiation of combination diuretic therapy)b. Initiation of intravenous diuretic (even a single dose) or vasoactive agent (e.g. inotrope, vasopressor, vasodilator)c. Mechanical or surgical intervention, including:i. Mechanical circulatory support (e.g. intra-aortic balloon pump, ventricular assist device, extracorporeal membrane oxygenation, total artificial heart)ii. Mechanical fluid removal (e.g. ultrafiltration, hemofiltration, dialysis)**General Considerations (HF Hospitalization)**Combination diuretic therapy could include (1) a thiazide-type diuretic (e.g. hydrochlorothiazide, metolazone, chlorothiazide) plus a loop diuretic; or (2) mineralocorticoid receptor antagonist (MRA) (e.g. spironolactone or eplerenone) plus a loop diuretic.
Heart Failure Collaboratory and Academic Research Consortium (2020)^a^	**Heart failure hospitalization**A sub-category of cardiovascular hospitalization.An admission to the hospital where patient length of stay extends for at least 24 h or as measured by a change in calendar date, and:i. The patient is admitted to the hospital with a primary diagnosis of HF.ii. The patient exhibits documented new or worsening symptoms due to HF on presentation, including at least one of the following: dyspnoea (dyspnoea with exertion, dyspnoea at rest, orthopnoea, PND); decreased exercise tolerance; fatigue; or other symptoms of worsened end-organ perfusion or volume overload that must be specified and described by the trial protocol.iii. The patient has objective evidence of new or worsening HF, consisting of at least two physical examination findings or one physical examination finding and one laboratory or invasively measured criterion, including:a. Physical examination findings considered to be due to HF include new or worsened: peripheral oedema; increasing abdominal distention or ascites (in the absence of primary hepatic disease); pulmonary rales/crackles/crepitations; increased JVP and/or hepatojugular reflux; S3 gallop; clinically significant or rapid weight gain thought to be related to fluid retention.b. Laboratory evidence of new or worsening HF, if obtained within 24 h of presentation, include:increased BNP/N-terminal pro B-type natriuretic peptide (NT-proBNP) concentrations consistent with decompensation of HF (in patients with chronically elevated natriuretic peptides, a significant increase should be noted above baseline); radiological, ultrasonographic, or implantable monitor evidence of pulmonary congestion; non-invasive or implantable diagnostic evidence of clinically significant elevated left- or right-sided ventricular filling pressure, or low cardiac output.c. Invasive evidence of new or worsening HF including right heart catheterization showing elevated pulmonary capillary wedge pressure (pulmonary artery occlusion pressure), elevated central venouspressure, depressed cardiac index, or left heart catheterization showing elevated left ventricular end diastolic pressure consistent with decompensation of HF.iv. The patient receives at least one of the following treatments specifically for HF:a. Significant augmentation in oral diuretic therapy (at least a doubling of loop diuretic dose, initiation of maintenance loop diuretic therapy, initiation of combination diuretic therapy).b. Initiation of intravenous diuretic (even a single dose).c. Initiation of an intravenous vasoactive agent (catecholamine, phosphodiesterase-3 inhibitor, other vasopressor, vasodilator).d. Mechanical or surgical intervention, including mechanical circulatory support (intra-aortic balloon pump, temporary, or durable ventricular assist device including both percutaneous and surgically placed devices, extracorporeal membrane oxygenation, or total artificial heart).e. Mechanical fluid removal (ultrafiltration, haemofiltration, haemodialysis).v. However, in cases where documentation of the above criteria may not be obtained, the following definition of a HF hospitalization may be used: If agreed by consensus of a clinical events committee, events of symptomatic HF that include at least one worsening symptom, as well as either one physical examination finding or one laboratory finding or invasively measured criterion, in addition to at least one intravenous or mechanical treatment specific for HF, may be considered HF hospitalizations, with or without supporting documentation.vi. Events that do not meet the criteria for any of the preceding definitions may be collected and analysed according to a pre-specified plan.

^a^Also includes definitions for **worsening heart failure in the setting of acute heart failure, worsening heart failure in the setting of a non-heart failure hospitalization**, and **worsening heart failure events without hospitalization.**

Our finding that heart failure events are not commonly reported in dialysis trials is surprising given their prognostic importance and frequency in kidney failure and the recommendation for standardized outcome reporting in dialysis [18]. In a systematic review of 362 haemodialysis trials, 44 of 362 reported CV disease but heart failure events were not collected [19]. In a systematic review of 120 peritoneal dialysis trials, 11 of 120 reported CV function and 1 of 120 reported a CV event as the primary outcome and none reported heart failure hospitalization as an outcome [20]. A systematic review of haemodialysis trials from 2011–2017 identified that 174 of 391 (44.5%) reported at least 1 CV outcome of which 52 (30%) included a CV composite outcome, of which 27 (30%) included acute coronary syndrome, hospitalization for heart failure, nonfatal stroke, or CV death [21]. This clearly appears to be an understudied area and given the rarity of heart failure event reporting in our meta-epidemiologic study, it certainly plausible that investigators may be avoiding selecting heart failure events as outcomes in their trials given the degree of uncertainty in their definition and ascertainment.

The SONG-HD consensus workshop (which included patients receiving haemodialysis, caregivers, and clinicians) on selecting, defining and implementing a core outcome measure for CV disease in trials in haemodialysis recommended using myocardial infarction as the core outcome for CV disease and sudden cardiac death as the core outcome measure for CV death given their prevalence, impact on quality of life, function and long-term outcomes, feasibility of measurement, and their potential to be modified by interventions and for transparency and accurate interpretation of individual non-composite outcomes. Regarding other core outcome measures, there was a recommendation for consistent standardized definitions that could be used in the context of routine clinical care and trials to align outcome ascertainment [22]. KDIGO also recommends the consideration of standardized disease outcome definitions that are feasible to apply at scale when available or appropriate (but not as core outcomes) and that new definitions may be necessary in special situations such as heart failure in dialysis where adjudication may materially influence the interpretation of results [23] as seen in the PIVOTAL trial. PIVOTAL is the largest contemporary RCT in dialysis with a heart failure event when detailed event adjudication and reporting with a total of 110 heart failure hospitalizations and 28 heart failure deaths in its 2141 participants (low heart failure events rates) that were reduced with adjudication as seen with other outcomes such as atherosclerosis events in SHARP [24]. With regards to heart failure hospitalizations, 80% had dyspnoea while only 40% had orthopnoea and only 19% had paroxysmal nocturnal dyspnoea (PND), 44% had pulmonary oedema while 76% had radiographical pulmonary oedema or congestion, and 29% had pulmonary oedema. An echocardiogram was carried out in 40% of cases (70% had left ventricular systolic dysfunction) and brain natriuretic peptide testing was rare (10%). The most common treatment was mechanical ultrafiltration (88%) while 15% received intravenous diuretics and <3% received other intensive therapies including vasodilators/vasopressor/inotropes or invasive/non-invasive ventilation [25]. Of note, due to a programming error, PIVOTAL's initial results were based on investigator reported events as a result of a programming error but this was subsequently updated to include only adjudicated events that resulted in fewer events and differences in effect sizes [17, 26].

### Heart failure versus volume overload

Volume overload and neurohormonal compensatory mechanisms involved in kidney failure can overlap with the symptoms [27], signs, haemodynamics, and cardiac biomarker profiles of heart failure making the distinction between the two challenging if not impossible. In these cases, the cause of the symptoms and signs of volume overload is a distinct entity to which treatment is often primarily directed, in addition to heart failure if is clinically apparent. Whether or not heart failure with reduced or preserved ejection fraction is present may help guide clinical decision making including additional investigation (e.g. imaging, angiography) and goal directed medical therapy. These events are often relevant to clinical events adjudication committees of RCTs, where events may be considered as an ‘events with heart failure’ rather than a ‘primary heart failure event’. An important concept that supports the differentiation of these competing diagnoses are that the symptoms and signs of heart failure may disappear once the underlying primary cause is treated, for example, symptoms and signs that mimic heart failure may resolve with dialysis if a patient may have missed one or more dialysis treatments or was non-adherent with salt/water restriction, which may also be the case in patients with heart failure without kidney disease [28]. Unfortunately, rigorously distinguishing the two entities is complicated by the high prevalence of abnormal cardiac structure and function in patients with kidney failure [29]. Diagnostic criteria have been proposed that attempt to distinguish those with heart failure that is or is not responsive to ultrafiltration [30] but most patients with CKD, and presumably kidney failure, would meet echocardiographic criteria proposed by Acute Dialysis Quality Initiative resulting in an overdiagnosis of heart failure without providing substantial additional prognostic information or change in clinical management [31]. Nevertheless, clinicians frequently make distinctions between heart failure and volume overload, and administrative data suggest that patients receiving dialysis with a hospitalization diagnosis of heart failure have substantially worse prognosis that those diagnosed with only fluid overload or pulmonary oedema [28]. This implies that valuable clinical distinctions are being made, although the clinical reasoning underlying this is yet to be formalized into a data driven definition with validated diagnostic criteria that can be practically implemented in clinical trial settings.

### Strengths and limitations

The strengths of this study include its size and inclusion of all contemporary RCTs in dialysis in top medical, nephrology, and cardiology journals over the last two decades, and its detailed data abstraction from primary studies, supplemental materials, protocols, and registrations. However, it has limitations. First, it only focused on high-impact journals to strike a balance between feasibility and generalizability (however, RCTs are less likely to be published in lower impact journals and trials published in high-impact journals are more likely to be well designed and impact medical practice). Second, it did not include any studies dedicated to paediatrics and CKD/transplant given the focus on adults and dialysis. Third, it lacks heart failure event definitions for peritoneal dialysis. Last, there was a lack of representation of trials from settings other than Asia, Europe, and North America where heart failure management and dialysis practices may vary [11].

### Recommendations

Given heart failure events are important to patients, care providers, regulators, and the health care system, there is a need for standardized diagnostic criteria. Our meta-epidemiologic systematic review shows that standardization of heart failure diagnostic criteria in dialysis is absent. However, there is an opportunity to derive diagnostic criteria of heart failure events that will improve the conduct of future RCTs to identify effective therapies. Such an endeavour will require explicit and transparent diagnostic criteria used in studies and collaboration across studies. In the interim, those designing RCTs for patients receiving dialysis should consider measuring heart failure events when relevant, and explicitly defining these events after considering existing diagnostic criteria and the issues discussed before here including what pathophysiology their treatment works on and even contacting other trial designers to discuss what has and has not been practical in operationalizing these diagnostic criteria. In addition, as standardized criteria are developed, information on heart function and structure from imaging studies done at baseline may help understand the aetiology of heart failure events and refine definitions over time.

Different RCTs have different purposes (e.g. mechanistic, early drug development, pivotal) and this may affect the specific components or diagnostic criteria for heart failure events. All diagnostic criteria for heart failure events should reflect their effect on morbidity (e.g. impaired quality of life, future health care utilization) and mortality. Recognizing this, the community may benefit from multiple or nested standardized diagnostic criteria for heart failure events that vary to correspond to available resources and the need for sensitivity or specificity to treatment effects and aetiology. This is analogous to stroke criteria where both ischaemic and haemorrhagic strokes require permanent focal neurologic deficits, but the ischaemic strokes are often separated from haemorrhagic on the basis of the likely stroke aetiology or supporting imaging or treatment [7]. Applying these concepts to patient with kidney failure is potentially complicated by issues such as residual kidney function, the prevalence of elevated cardiac biomarker concentrations [32], the sensitivity of echocardiography to volume status and timing relative to dialysis treatments [33], the interdialytic interval [34], and the roles of lung ultrasound [35] and bioimpedance spectroscopy [36]. Comparing the consistency of effect sizes based on investigator reported events and adjudicated events across conservative and liberalized diagnostic criteria may also prove to be insightful. Despite the (iterative) work required to establish standardized diagnostic criteria such an endeavour is likely to have a lasting positive impact on the conduct of RCTs in patients receiving dialysis and ultimately the generation of evidence that improves outcomes.

In summary, heart failure events are infrequently reported in RCTs in dialysis and have heterogeneously diagnostic criteria according to symptoms, signs, laboratory tests, imaging, health care utilization, and interventions without any distinguishment from volume overload. Further research is required to develop a standardized, practical, and robust diagnostic criteria to differentiate heart failure from volume overload.

## Data Availability

The data underlying this article will be shared on reasonable request to the corresponding author.
